# Distinguishing heart failure subtypes: the diagnostic power of different cardiac magnetic resonance imaging parameters

**DOI:** 10.3389/fcvm.2024.1291735

**Published:** 2024-02-07

**Authors:** Yanhui Hao, Rui Zhang, Lihong Chen, Ganglian Fan, Bing Liu, Ke Jiang, Yi Zhu, Ming Zhang, Jianxin Guo

**Affiliations:** ^1^ Department of Radiology, The First Affiliated Hospital of Xi’an Jiaotong University, Xi’an, China; ^2^ Clinical & Technical Support, Philips Healthcare, Beijing, China

**Keywords:** cardiac magnetic resonance imaging (CMR), heart failure (HF), myocardial strain, extracellular volume fractions (ECV), T1 mapping

## Abstract

**Objectives:**

The aim of this retrospective study was to explore the diagnostic potential of various cardiac parameters in differentiating between heart failure with preserved ejection fraction (HFpEF) and heart failure with mid-ranged and reduced ejection fraction (HFm + rEF), and to discern their relationship with normal cardiac function.

**Methods:**

This research encompassed a comparative analysis of heart failure subtypes based on multiple indicators. Participants were categorized into HFm + rEF, HFpEF, and control groups. For each participant, we investigated indicators of left ventricular function (LVEDVi, LVESVi, and LVEF) and myocardial strain parameters (GLS, GCS, GRS). Additionally, quantitative tissue evaluation parameters including native T1, enhanced T1, and extracellular volume (ECV) were examined.For comprehensive diagnostic performance analysis, receiver operating characteristic (ROC) curve evaluations for each parameters were conducted.

**Results:**

HFm + rEF patients exhibited elevated LVEDVi and LVESVi and decreased LVEF compared to both HFpEF and control groups. Myocardial strain revealed significant reductions in GLS, GCS, and GRS for HFm + rEF patients compared to the other groups. HFpEF patients showed strain reductions relative to the control group. In cardiac magnetic resonance imaging (CMR) evaluations, HFm + rEF patients demonstrated heightened native T1 times and ECV fractions. Native T1 was particularly effective in distinguishing HFpEF from healthy subjects.

**Conclusion:**

Native T1, ECV, and myocardial strain parameters have substantial diagnostic value in identifying HFpEF. Among them, native T1 displayed superior diagnostic efficiency relative to ECV, offering critical insights into early-stage HFpEF. These findings can play a pivotal role in refining clinical management and treatment strategies for heart failure patients.

## 1 Introduction

Heart Failure (HF) is a critical clinical condition caused by cardiac structural and functional abnormalities, leading to high mortality and rehospitalization rates, and becoming one of the major diseases threatening human health (1).

The American Heart Association, the American College of Cardiology, and the Heart Failure Society of America jointly released the revised “2022 AHA/ACC/HFSA Heart Failure Management Guidelines,” classifying HF patients into four types based on left ventricular ejection fraction (LVEF) (2). Among these, heart failure with preserved Ejection Fraction (HFpEF) accounts for over half of HF patients, and early intervention in HFpEF can reduce morbidity and mortality (3).

Diagnosing HFpEF is challenging due to its diverse etiology and phenotype (4, 5). Recent studies have shown that myocardial fibrosis and increased diastolic wall stiffness, leading to elevated left ventricular filling pressures, are central to the pathophysiology of HFpEF (6, 7).

The current “gold standard” for diagnosing HFpEF is exercise right heart catheterization, an invasive procedure (8). Therefore, non-invasive imaging examinations and diagnostics are crucial in understanding the changes in cardiac structure and function in HFpEF patients. Cardiac magnetic resonance (CMR) offers high spatial resolution and a broad view of cardiac structure and has become indispensable for accurately diagnosing and stratifying heart failure patients. Recent advancements in specific cardiac MRI techniques have introduced new dimensions to our understanding and diagnosis of heart failure.

Assessment of the left ventricular function, including parameters like left ventricular end-diastolic volume index (LVEDVi), left ventricular end-systolic volume index (LVESVi), left ventricular ejection fraction (LVEF), stroke volume (SV), and cardiac output (CO), is crucial in HFpEF patients. Abnormalities in these metrics can reflect the functional changes in the heart, highlighting the significance of their assessment in clinical settings.

Beyond left ventricular function, some quantitative tissue evaluation parameters, such as native T1 mapping, enhanced T1 mapping, and extracellular volume (ECV), have garnered significant attention (9). These parameters assist in characterizing the underlying myocardial tissue properties. Native T1 mapping and enhanced T1 mapping, representing non-contrast and contrast-based T1 relaxation time measurements respectively, hold significant promise. In heart failure contexts, these techniques capture changes in the myocardium that may indicate early or evolving myocardial diseases, even before overt heart failure symptoms manifest (10, 11). They provide a window into the cellular and extracellular matrix alterations that often precede clinical heart failure (12). The ECV quantifies the extracellular space in myocardial tissues. Given the association of myocardial fibrosis with heart failure, especially in its diastolic form, ECV's ability to gauge fibrotic changes in the myocardium aids in the early detection and monitoring of heart failure progression.

HFpEF was previously considered a type of diastolic heart failure. Recent research has revealed multifactorial interactions between increased left ventricular (LV) stiffness due to elevated left ventricular diastolic pressure (LVDP), abnormalities in both systolic and diastolic function, and metabolic abnormalities (13). Multiple studies have shown a reduction in myocardial strain in the systolic left ventricular wall of HFpEF patients (14–16). Thus, by evaluating cardiac response to stress, myocardial stress testing can identify areas of the heart with compromised perfusion or subtle dysfunctions.

Due to the high hospitalization rate and poor prognosis of HFpEF patients, and the fact that most existing HF drugs are less effective in HFpEF than in heart failure with mid-ranged and reduced ejection fraction (HFm + rEF), a comprehensive understanding of HFpEF can not only improve diagnostic accuracy but also lead to new therapeutic approaches. The objective of this study is to synthesize the exploration of CMR parameters such as LVEDVi, LVESVi, LVEF, SV, CO, naive T1, enhanced T1, ECV, and myocardial strain, and to identify the most effective indicators for the diagnosis of HFpEF, thus providing the scientific basis for physicians to formulate appropriate treatment plans.

## 2 Materials and methods

### 2.1 Study population

This retrospective study included consecutive patients with heart failure who underwent CMR and echocardiography at our hospital between January 2021 and July 2022. Ethical approval was obtained from the ethics committee of The First Affiliated Hospital of Xi'An Jiao Tong University, and written informed consent was waived due to the retrospective nature of the study.

In this study, subjects were stratified into three distinct groups in accordance with contemporary guidelines (17). The classification criteria were as follows:

(1) HFpEF: This category included patients exhibiting an ejection fraction (EF) ≥ 50% coupled with brain natriuretic peptide level exceeding 35 pg/ml or an N-terminal pro-brain natriuretic peptide level surpassing 125 pg/ml at the point of diagnosis. Additionally, these patients demonstrated at least one of the subsequent conditions: (a) structural abnormalities in the left ventricle, as evidenced by a left ventricular end-diastole mass index greater than 115 g/m^2^ in males and over 95 g/m^2^ in females, determined via CMR; or (b) signs of left ventricular diastolic dysfunction, characterized by an early and/or late peak diastolic mitral inflow velocity (E/A ratio) lower than 1, as assessed by echocardiography.
(2) HFm + rEF: Patients in this group were identified by an EF ranging from 40%–49% or a markedly reduced EF below 40%. Apart from the specific EF thresholds, these patients adhered to similar diagnostic criteria as the HFpEF group, including the same natriuretic peptide levels and evidence of either left ventricular structural abnormalities or diastolic dysfunction.
(3) Normal Control Group: This group comprised individuals who attended routine health screenings. These participants had no cardiovascular risk factors such as hypertension, diabetes, or hyperlipidemia, were not on any cardiac medications, and displayed normal results in CMR imaging, 12-lead electrocardiography, and echocardiography assessments.

**Figure 1:**
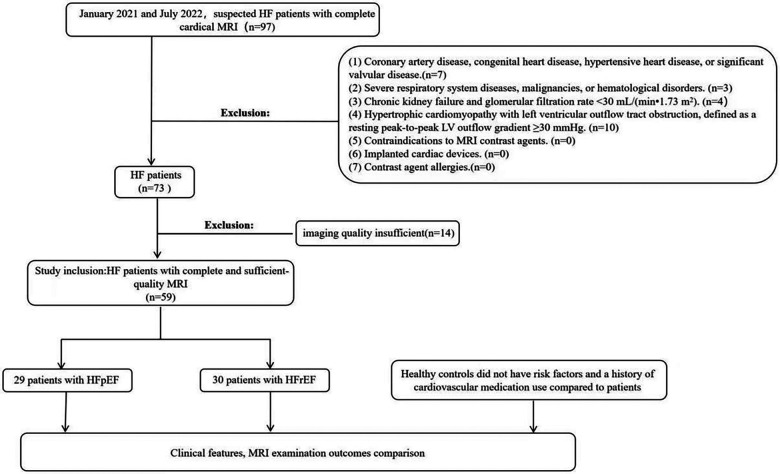
Partipant flowchart. LV, left ventricular; HF, heart failure; HFpEF, heart failure with preserved ejection fraction.

Exclusion criteria included: (1) Patients with coronary artery disease confirmed by coronary computed tomography angiography or coronary angiography, congenital heart disease, hypertensive heart disease, or significant valvular disease. (2) Patients with severe respiratory system diseases, malignancies, or hematological disorders. (3) Patients with chronic kidney failure and glomerular filtration rate <30 ml/(min·1.73 m^2^). (4) Patients with hypertrophic cardiomyopathy with left ventricular outflow tract obstruction, defined as a resting peak-to-peak LV outflow gradient ≥30 mmHg. (5) Patients with contraindications to MRI contrast agents. (6) Patients with implanted cardiac devices. (7) Patients with contrast agent allergies. (Figure 1).

### 2.2 Cardiovascular MRI acquisition

The CMR imaging was conducted employing a Philips 3.0 T MR scanner (IngeniaCX, Philips, The Netherlands). For contrast enhancement, Gadobutrol Injection, a gadolinium-based contrast agent, was utilized. Long and short-axis views of the left ventricle (LV) were captured using retrospectively gated steady-state free precession (SSFP) cine sequences. The acquisition parameters were set as follows: repetition time (TR) at 2.7 ms, echo time (TE) at 1.34 ms, flip angle (FA) at 45°, and a voxel size of 2.5 × 2.5 × 8 mm³. The field of view (FOV) was standardized at 300 × 300. Both native and post-contrast enhanced T1 mapping were executed in basal, mid, and apical short-axis slices of the LV. This was achieved using a modified look-locker inversion recovery sequence. For the native T1 mapping, the parameters were TR of 2.2 ms, TE of 1.00 ms, FA of 20°, voxel size of 1.97 × 2.00 × 10.0 mm³, and an FOV of 300 × 300. Similarly, the enhanced T1 mapping was performed with TR set at 2.1 ms, TE at 0.98 ms, FA at 20°, voxel size at 1.97 × 2.00 × 10.0 mm³, and the same FOV of 300 × 300. Each slice's acquisition spanned approximately 12 s.

### 2.3 Cardiovascular MRI analysis

**Figure 2:**
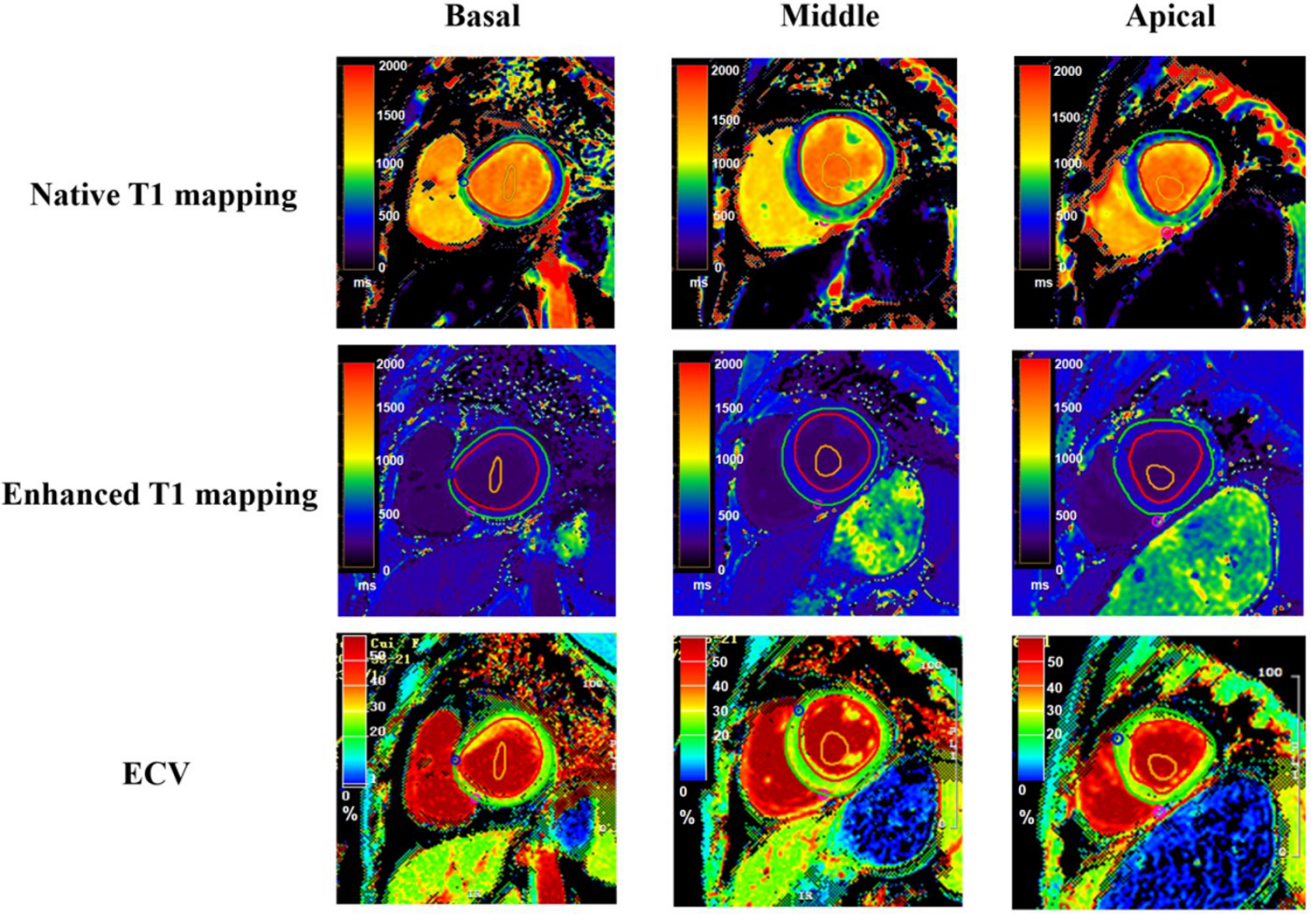
T1 mapping and ECV quantification in left ventricular segmentation. This figure displays T1 mapping in the basal (left column), mid-ventricular (middle column), and apical (right column) short-axis segments of the left ventricle from a single patient. The top row illustrates native T1 maps, the middle row shows post-contrast T1 maps, and the bottom row depicts the calculated extracellular volume (ECV) maps for corresponding segments. The red region of interest represents the epicardium, the green region of interest represents the endocardium, and the orange region of interest represents the blood pool.

Image analysis was conducted utilizing the commercial software package CVI42 (Circle Cardiovascular Imaging Inc., Calgary, AB, Canada). An experienced reader, blind to the patient's clinical history, other CMR images, and clinical outcomes, performed manual delineation of the endocardial and epicardial borders in a randomized order for the basal, mid-cavity, and apical segments of the left ventricle (Figure 2). The analysis excluded the 17-segment heart apex model of the American Heart Association (AHA), and primary T1 values and post-contrast values were calculated from the remaining 16 regions (18). Additionally, global average T1 values were assessed.

For each basal, middle, and apical myocardial segment, myocardial ECV was derived using pre- and post-contrast T1 values of the myocardium and blood pool, following previously described methods (19). Hematocrit levels (Hct) were obtained through a blood draw within three days before the MRI examination. Briefly, ECV was calculated as (1−Hct) × (*Δ*R1myocardium/*Δ*R1blood), where Hct is the hematocrit level, and *Δ*R1 represents the change in T1 relaxivity (R1 = 1/T1) before and after gadolinium-based contrast administration.

### 2.4 Assessing the cardiac volume and function

Images were analyzed offline using the short 3D module with the CVI42 software (CVI42; Circle Cardiovascular Imaging Inc., Calgary, AB, Canada). Left ventricular enddiastolic volume index, left ventricular end-systolic volume index, stroke volume, cardiac output and left ventricular ejection fraction were measured in the short axis the short axis.

### 2.5 Myocardial strain analysis

Myocardial strain analysis was performed on the short-axis and long-axis (2Ch, 3Ch, and 4Ch) cine images in CVI42 software. The software automatically tracked the myocardial motions in each cardiac cycle. Endocardial and epicardial boundaries were traced semiautomatically, with manual corrections applied as necessary, particularly in cases of inaccurate contour recognition or pooradaptability of the automated tracing. Three-directional myocardial strains, including global longitudinal strain (GLS), global radial strain (GRS), and global circumferential strain (GCS), were calculated according to the AHA 17-segment model, with the apex excluded.

### 2.6 Statistical analysis

Statistical evaluation of the data was carried out utilizing the GraphPad Version 10.0 software. The Shapiro–Wilk test was used to assess the normality of continuous variables. Normally distributed data were presented as mean ± standard deviation (x ± s), while non-normally distributed data were presented as median (interquartile range). For normally distributed continuous variables, independent sample *t*-tests were used for comparisons between two groups, and one-way ANOVA was used for comparisons among three groups. Non-parametric tests were used for non-normally distributed continuous variables. Count data were presented as percentages and analyzed using the chi-square test. Receiver operating characteristic (ROC) curves were generated to evaluate the area under the curve (AUC) for the best cutoff point. The Delong test was used to evaluate the significance of the differences between the ROC curves. A *P*-value of less than 0.05 was considered indicative of statistical significance.

## 3 Results

### 3.1 Characteristics of healthy controls and patients

The study comprised 30 patients with HFm + rEF, 29 with HFpEF, and 17 healthy controls without HF. Table 1 encapsulates the fundamental characteristics of these subjects. Across these groups, variables such as age, sex, blood pressure, and body mass index (BMI) showed no significant disparities. In contrast to patient groups, the healthy control subjects were characterized by an absence of risk factors and a lack of history with cardiovascular medications.

**Table 1:** The clinical characteristics of the study population.

	Controls (*n* = 17)	HFpEF (*n* = 29)	HFm + rEF (*n* = 30)	*P*
Age(year)	52 ± 17	52 ± 12	51 ± 15	0.9
Male, *n* (%)	11 (55)	11 (38)	20 (67)	0.2
Body mass index (kg/m^2^)	24.0 ± 2.8	25.7 ± 3.5	25.1 ± 2.3	0.8
Systolic blood pressure (mm Hg)	116 (107–123)	129 (113–139)	112 (101–130)	0.5
Diastolic blood pressure (mm Hg)	73 (69–86)	78 (71–85)	71 (64–91)	0.3
Risk factors *n* (%)				
History of hypertension	0	14 (48)	8 (27)	0.02
History of dyslipidemia	0	2 (7)	5 (17)	0.1
History of diabetes	0	4 (14)	4 (13)	0.2
History of myocardial infarction	0	5 (17)	7 (23)	0.07
Medications *n* (%)				
Atrial fibrillation	0	3 (10)	2 (7)	0.3
ACE inhibitors or ARBs	0	11 (38)	14 (47)	0.6
Loop diuretics	0	4 (14)	6 (20)	0.7
Calcium channels-blockers	0	8 (28)	10 (33)	0.8
Beta-blockers	0	6 (21)	12 (40)	0.2
Oral antiglycaemic agents	0	5(17)	7(23)	0.7

HFpEF, heart failure with preserved ejection fraction; HFm + rEF, heart failure with mid-ranged and reduced ejection fraction; ACE inhibitors, angiotensin-converting enzyme inhibitors; ARBs, angiotensin II receptorblockers.

### 3.2 LV functional parameters from CMR

**Figure 3:**
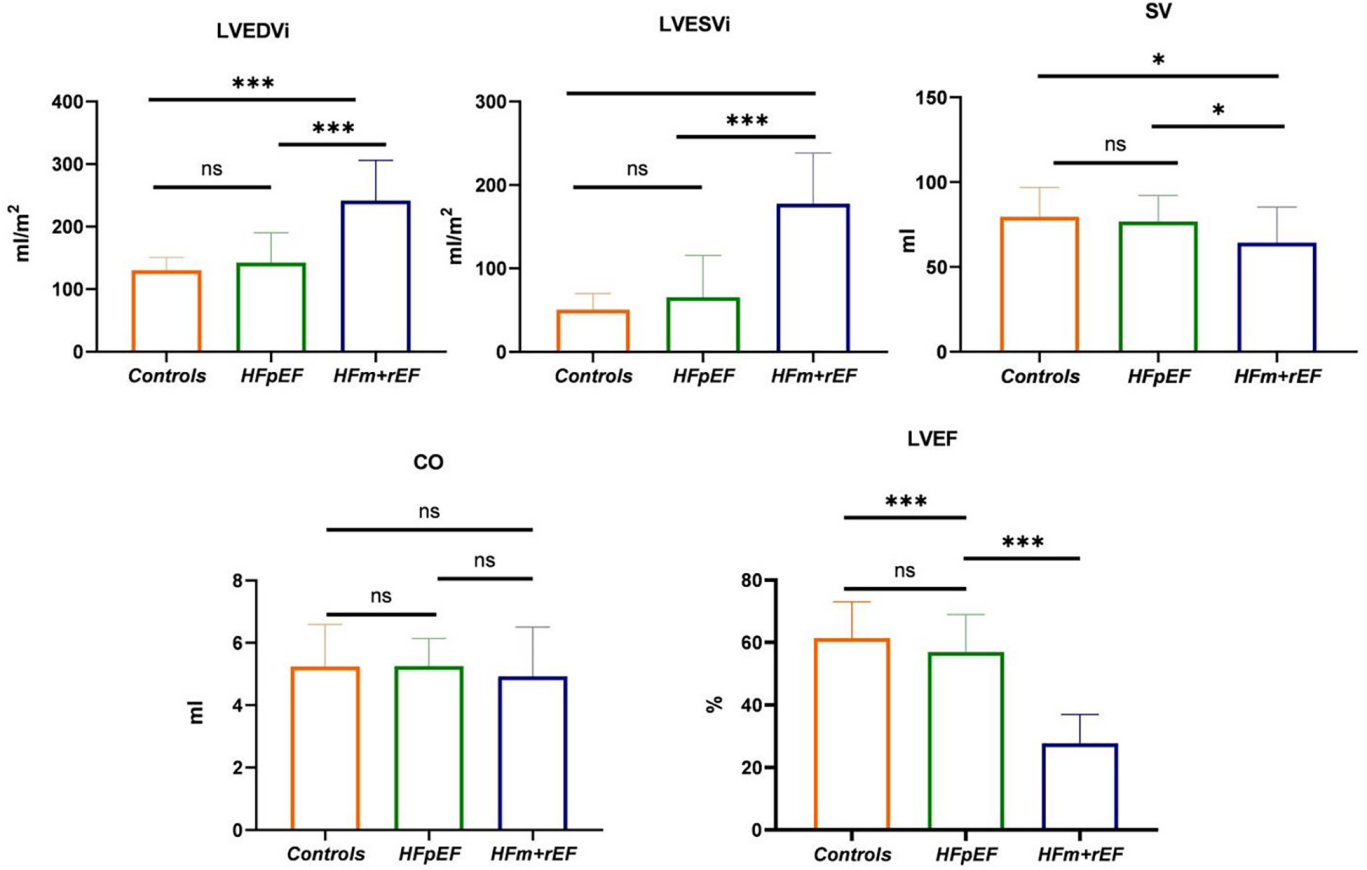
Group-Wise comparison of CMR imaging metrics using Bar graphs.the bars represent the mean values for each group, and the error bars indicate the standard deviation. This bar graph depicts the collective CMR imaging metrics for healthy control, and patients diagnosed with HFpEF or HFm + rEF. Displayed metrics include LVEDVi, LVESVI and SV on the top row, LVEF and GLS on the bottom row.

Table 2 presents a comparative analysis of MRI parameters across three groups. There are significant differences were observed between the HFm + rEF and HFpEF groups. Patients with HFm + rEF exhibited elevated left ventricular functional parameters, including an increased left ventricular end-diastolic volume index (LVEDVi; 242 ± 64 ml/m^2^ vs. 141 ± 48 ml/m^2^, *P* < 0.01) and a higher left ventricular end-systolic volume index (LVESVi; 178 ± 61 ml/m^2^ vs. 65 ± 51 ml/m^2^, *P* < 0.01). Furthermore, a reduced stroke volume was noted in the HFm + rEF group compared to the HFpEF group (64 ± 21 ml vs. 76 ± 15 ml, *P* < 0.01). When compared to the control group, the HFm + rEF group also showed elevated left ventricular functional parameters, with increased LVEDVi (242 ± 64 ml/m^2^ vs. 130 ± 20 ml/m^2^, *P* < 0.01) and LVESVi (178 ± 61 ml/m^2^ vs. 51 ± 19 ml/m^2^, *P* < 0.01), along with a decreased stroke volume (64 ± 21 ml vs. 80 ± 17 ml, *P* < 0.01). However, no significant statistical differences were observed in the left ventricular functional parameters between the HFpEF and control groups. Additionally, CO and septal thickness did not significantly differ among all three groups (HFpEF, HFm + rEF, and controls). (Table 2; Figure 3).

**Table 2:** The clinical characteristics of the study population.

	Controls (*n* = 17)	HFpEF (*n* = 29)	HFm + rEF (*n* = 30)	*P*
LVEDVi (ml/m^2^)	130 ± 20	141 ± 48	242 ± 64	<0.01
LVESVi (ml/m^2^)	51 ± 19	65 ± 51	178 ± 61	<0.01
SV (ml)	80 ± 17	76 ± 15	64 ± 21	<0.01
CO (ml)	5.3 ± 1.4	5.27 ± 0.90	4.9 ± 1.6	>0.05
LVEF (%)	61 ± 12	57 ± 13	27.7 ± 9.3	<0.01
Apical septum (mm)	9.4 ± 2.2	11.1 ± 3.1	10.2 ± 3.1	>0.05
Mid septum (mm)	11.0 ± 2.1	12.8 ± 3.0	12.7 ± 4.3	>0.05
Basal septum (mm)	10.3 ± 2.4	12.0 ± 3.0	10.5 ± 2.9	>0.05
ECV (%)	27.9 ± 5.3	34.3 ± 5.4	37.3 ± 5.4	<0.05
Native T1 (ms)	1,283 ± 43	1,379 ± 79	1,430 ± 99	<0.01
Enhance T1 (ms)	448 ± 70	423 ± 63	411 ± 84	>0.05
GCS (%)	−20.3 ± 2.7	−15.8 ± 4.4	−7.5 ± 2.1	<0.01
GRS (%)	−17.7 ± 2.5	−14.2 ± 4.0	−7.9 ± 2.8	<0.01
GLS (%)	30.7 ± 6.2	24.3 ± 8.9	11.0 ± 3.3	<0.01

HFpEF, heart failure with preserved ejection fraction; HFm + rEF, heart failure with mid-ranged and reduced ejection fraction; LVEDVi, left ventricular end-diastolic volume index; LVESVi, left ventricular end-systolic volume index; SV, stroke volume; CO, cardiac output; LVEF, left ventricular ejection fraction; LVMi, left ventricular mass index; ECV, extracellular volume; GCS, global circumferential strain; GRS, global radial strain; GLS, global longitudinal strain.

### 3.3 Native T1, enhanced T1, ECV and myocardial strain parameters from CMR

**Figure 4:**
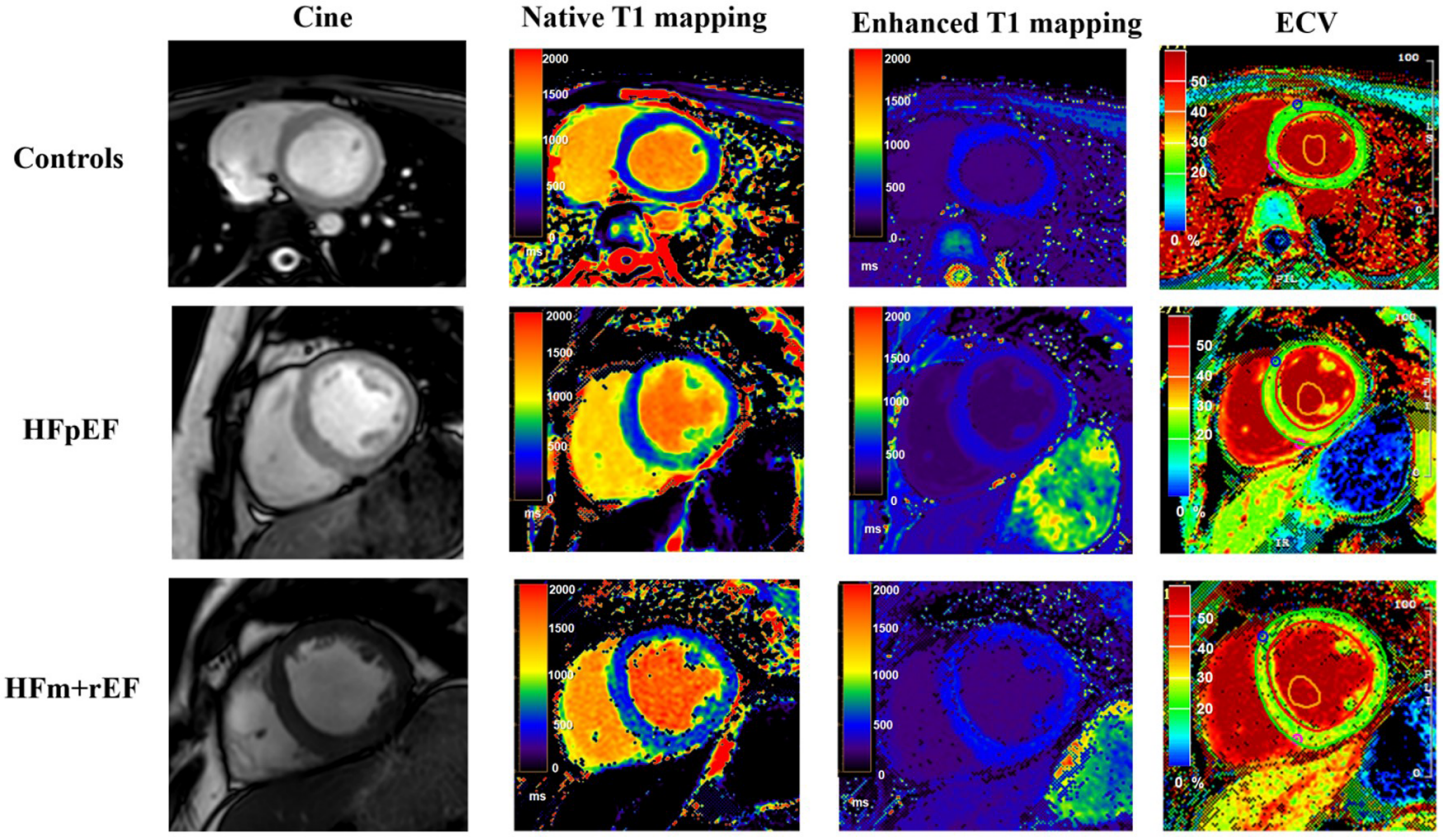
Comparative CMR imaging across patient groups. This figure presents a sequence of images for each group: control subjects, patients with Heart Failure with Preserved Ejection Fraction (HFpEF), and patients with Heart Failure with mid-range and reduced Ejection Fraction (HFm + rEF). From left to right, the images display cine, native T1 mapping, enhanced T1 mapping, and extracellular volume (ECV) measurement. The top row illustrates the control group, followed by the HFpEF patients in the middle row, and the HFm + rEF patients in the bottom row.

**Figure 5:**
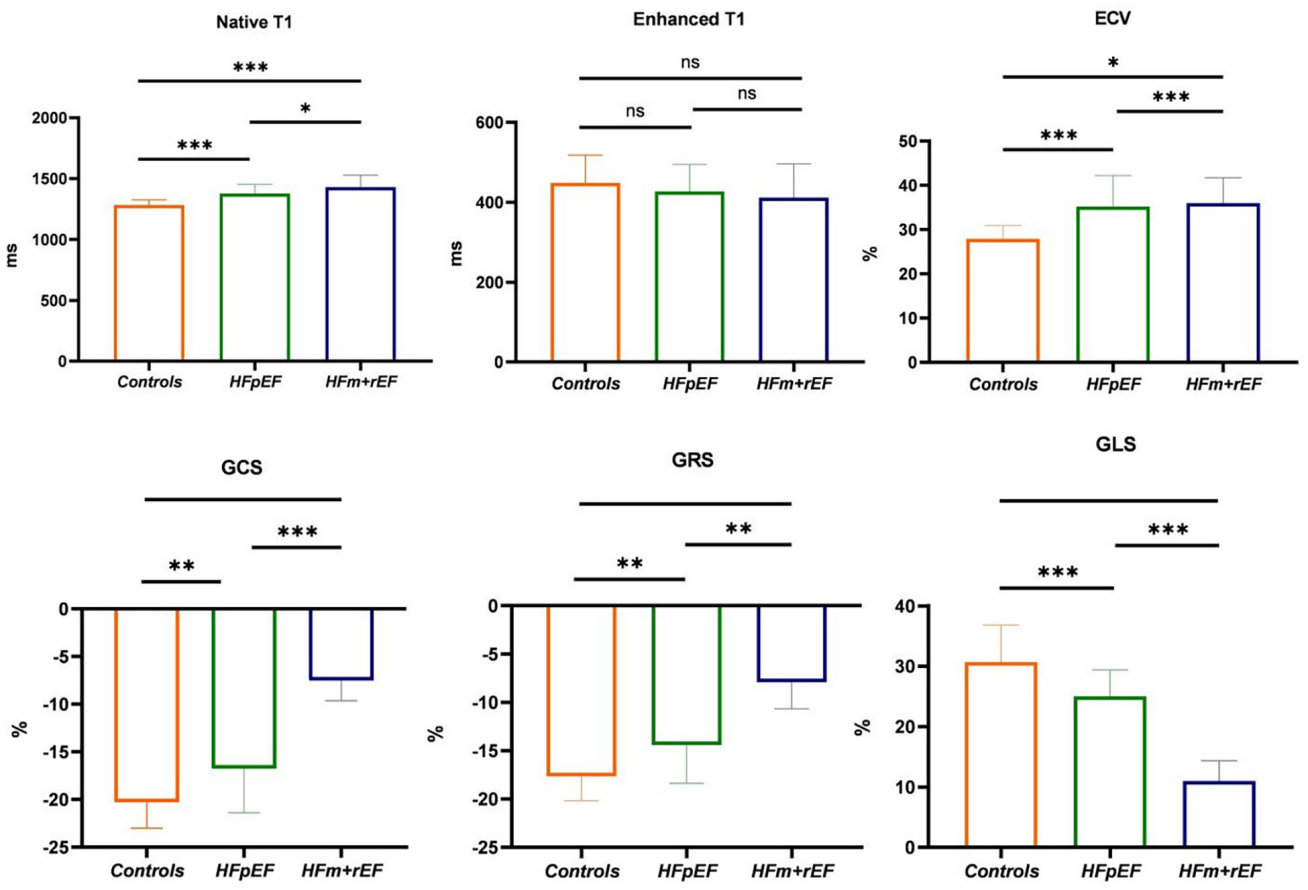
Group-Wise comparison of CMR imaging metrics using Bar graphs. The bars represent the mean values for each group, and the error bars indicate the standard deviation. This bar graph depicts the collective CMR imaging metrics for healthy control, and patients diagnosed with HFpEF or HFm + rEF. Displayed metrics include Native T1, Enhanced T1, and ECV on the top row, and myocardial strain indices: GCS, GRS, and GLS on the bottom row.

Compared with the HFpEF groups, the patients with the HFm + rEF showed higher myocardial ECV fractions(37.3 ± 5.4% vs. 34.3 ± 5.4%, *P* < 0.05), and native T1 times (1,430 ± 99 ms vs. 1,379 ± 79 ms, *P* < 0.05), no significant difference was detected in Enhanced T1 between patients with HFpEF and HFm + rEF. Compared with the controls groups, the patients with the HFm + rEF and HFpEF also showed larger ECV and native T1(37.3 ± 5.4% vs. 34.3 ± 5.4% vs. 27.9 ± 5.3%, *P* < 0.05; 1,430 ± 99 ms vs. 1,379 ± 79 ms vs. 1,283 ± 43 ms, *P* < 0.05) no significant difference was detected in enhanced T1 between patients with controls and HFpEF and HFm + rEF. Compared with the HFpEF groups, the patients with the HFm + rEF showed lower GCS (−15.8 ± 4.4% vs. −7.5 ± 2.1%, *P* < 0.01), the patients with the controls showed higher GCS (−15.8 ± 4.4% vs. −20.3 ± 2.7%, *P* < 0.01). GRS and GLS showed the same trend (Table 2; Figures 4, 5).

### 3.4 Differentiating HFpEF patients from normal controls

**Figure 6:**
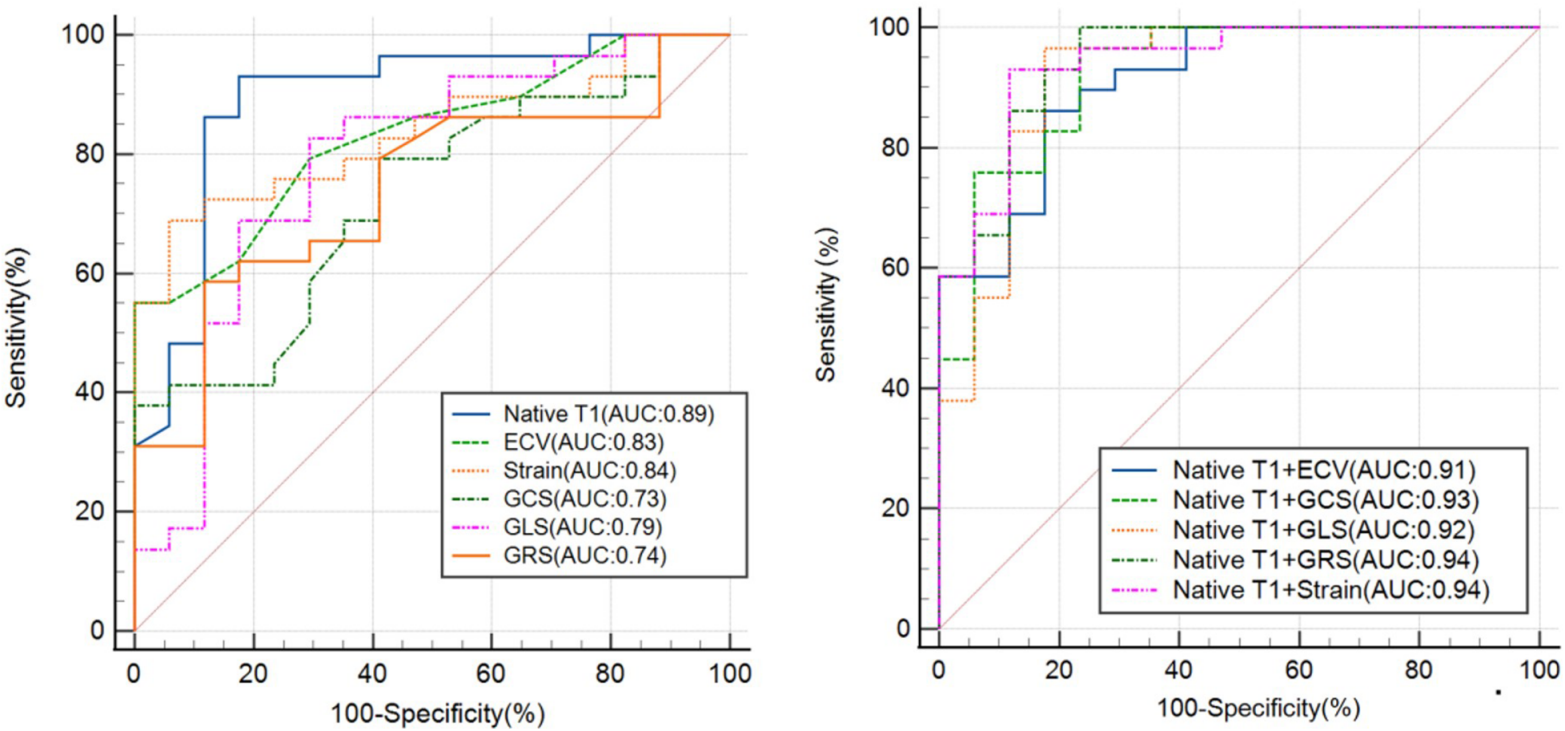
The ROC curve analysis of the CMR-derived LV parameters for differentiating patients with hFpEF from healthy controls. Strain refers to the combination of GLS, GRS and GCS. ROC, receiver operating characteristic; AUC, area under ROC curve; ECV, extracellular volume; GRS, global radial strain; GCS, global circumferential strain; GLS, global longitudinal strain.

The efficacy of T1 mapping and myocardial strain indices in differentiating patients with HFpEF from normal controls was evaluated (refer to Figure 6). Native T1 times demonstrated superior diagnostic accuracy with the highest area under the receiver operating characteristic curve (AUC-ROC) of 0.89, significantly surpassing the ECV fractions (AUC-ROC of 0.83, *p* < 0.05). A threshold of 1,302 ms for native T1 times effectively discriminated HFpEF patients from normal individuals with a sensitivity of 93% and a specificity of 82%. An ECV cut-off of 33% identified HFpEF patients with a sensitivity of 55% and a specificity of 100%, yielding an AUC-ROC of 0.83.

For myocardial strain indices, a cut-off of −19.6 for GCS differentiated HFpEF patients with a sensitivity of 79% and a specificity of 59% (AUC-ROC 0.73). A GRS threshold of −15.4 offered a sensitivity of 59% and a specificity of 88%, with an AUC-ROC of 0.74. A GLS cut-off of 28.3 provided a sensitivity of 83% and a specificity of 71%, with an AUC-ROC of 0.79. The combination of these three myocardial strain parameters yielded a higher AUC-ROC of 0.84, a sensitivity of 69%, and a specificity of 94%.

Integrating native T1 with strain indices enhanced the diagnostic performance. Combining native T1 and strain parameters, HFpEF patients were identified with a sensitivity of 93% and specificity of 88%, achieving an AUC-ROC of 0.94. The combination of native T1 and ECV distinguished HFpEF patients with a sensitivity of 86% and specificity of 82% (AUC-ROC 0.91). Merging native T1 and GCS, the sensitivity increased to 97% and specificity to 76%, with an AUC-ROC of 0.93. The amalgamation of native T1 and GLS resulted in a sensitivity of 97% and specificity of 82%, with an AUC-ROC of 0.92. Finally, combining native T1 with GRS achieved a sensitivity of 100% and specificity of 76%, with an AUC-ROC of 0.94.

## 4 Discussion

In the domain of heart failure diagnosis, CMR functional parameters are continuously being explored and investigated. This article delves deeply into a variety of CMR functional parameters, including left ventricular function parameters, myocardial strain parameters and quantitative tissue evaluation parameters, assessing their performance and manifestations in heart failure diagnosis. These findings not only expand our understanding of heart failure, particularly the distinctions between HFpEF and HFm + rEF, but also elucidate their relationship with normal cardiac function.

In this study, we initially centered our focus on indicators of left ventricular function, such as LVEDVi, LVESVi, and LVEF.n the present study, the HFm + rEF group demonstrated notably elevated LVEDVi and LVESVi values when compared to both the HFpEF group and the control group. However, no significant differences were observed between the HFpEF and control groups. Conversely, the LVEF values in the HFm + rEF group were significantly reduced compared to the HFpEF and control groups. From these findings, it is evident that left ventricular function parameters, namely LVEF, LVEDVi, and LVESVi, effectively differentiate between HF and the normal population. Nonetheless, they are not adept at distinguishing the normal population from HFpEF, aligning with previous studies (20, 21). A similar trend was evident for SV, while CO differences were not statistically significant among the three groups. The left ventricular functional parameters of the HFpEF group were found to be similar to those of the control group, whereas significant statistical differences were noted in the left ventricular functional parameters of the HFm + rEF group compared to the control group. Upon further examination of left ventricular structural parameters, it was noted that the HFpEF group exhibited a slightly increased wall thickness, but there was no statistically significant difference among the three groups, consistent with prior research. In HFm + rEF patients, who often present with dilated cardiomyopathy, eccentric remodeling occurs due to myocardial cell damage and loss, resulting in normal functional parameters but unchanged or thinned left ventricular walls. In contrast, HFpEF patients display notable clinical heterogeneity, including both concentric remodeling with increased wall thickness and normal or even eccentric patterns. This observation aligns with our study's findings (22, 23).

To delve deeper into resolving this quandary, we shifted our attention to parameters related to myocardial strain. Prior research has highlighted myocardial strain as a more sensitive early marker than LVEF functional parameters in heart failure, with myocardial stress alterations preceding changes in left ventricular function parameters (24). This study found that LVEF, LVEDVi, LVESVi, SV, and CO were not adept at discerning between the normal population and HFpEF. Consequently, we explored parameters associated with myocardial stress. Results indicated significant reductions in GLS, GCS, and GRS for HFm + rEF patients when compared to both HFpEF patients and healthy controls. Moreover, the HFpEF group demonstrated substantial reductions in these parameters compared to the control group. All three myocardial strain metrics—GCS, GLS, and GRS—exhibited robust diagnostic performance, with a combined OC-ROC of 0.84, sensitivity of 86%, and specificity of 70%. The combined AUC-ROC (0.84) was markedly superior to singular stress parameters and left ventricular function parameters. Previous investigations have corroborated the enhanced sensitivity of myocardial stress and stress rate in detecting subclinical cardiovascular diseases (25, 26). This study's findings further emphasize the utility of myocardial stress parameters as referential indicators for cardiac dysfunction, especially when EF values show negligible changes in the disease's early stages.

Non-contrast enhanced longitudinal relaxation time quantitative imaging (T1 mapping) is an efficient scanning technique that forgoes the need for contrast agents, emerging as an innovative CMR technology for the quantitative assessment of myocardial fibrosis (27). T1 values can be ascertained from native myocardium (native T1) or post-intravenous administration of gadolinium-based contrast agents (enhanced T1) (28, 29). The native T1 value represents the T1 characteristics of the myocardium and extracellular matrix and typically demonstrates prolonged relaxation times under pathological conditions like edema and fibrosis. Enhanced T1 values can detect diffuse fibrosis, providing precision in differentiating hypertrophic cardiomyopathy from fibrosis in non-ischemic cardiomyopathies (24). Moreover, native T1 characterization of myocardial tissue facilitates the detection and evaluation of various cardiomyopathies (30). Concurrently, T1 measurements with extracellular gadolinium-based contrast agents yield insights into the ECV fraction, reflecting the portion of myocardial tissue unoccupied by cardiomyocytes and thereby providing a quantitative measure of myocardial interstitial fibrosis. Histological evidence from previous research firmly establishes the close association between T1 mapping and ECV with myocardial fibrosis (31, 32). In the current study, HFm + rEF patients displayed significant elevations in native T1 times and ECV fractions in comparison to HFpEF patients, indicating heightened myocardial fibrosis in HFm + rEF patients. Elevated native T1 times can be ascribed to increased myocardial fibrosis or other pathological manifestations like edema, influenced by both intracellular and extracellular determinants. Importantly, both native T1 times and ECV fractions for HFpEF patients were heightened compared to healthy controls, suggesting their efficacy in distinguishing HFpEF from healthy individuals in instances of normal ejection fractions. The results of quantitative tissue evaluation parameters underscored the superiority of native T1 times in differentiating HFpEF patients from normal subjects, with a sensitivity of 97%, specificity of 75%, and a decisive AUC-ROC of 0.85 using a threshold value of 1,302 ms. In contrast, ECV demonstrated a sensitivity of 55%, specificity of 80%, and an AUC-ROC of 0.73 when distinguishing between healthy individuals and the HFpEF group. Notably, there was no discernible difference in enhanced T1 values across the three groups, possibly due to the pronounced variance in enhanced T1 values in widespread diffuse myocardial fibrosis. Since ECV values derive from enhanced T1 values, this might elucidate the comparatively reduced diagnostic efficacy of ECV relative to native T1.

While these findings offer novel perspectives and tools for the diagnosis of heart failure, our study is not devoid of limitations. Primarily, the absence of endomyocardial biopsy evaluations meant that we couldn't provide histological evidence to support the changes in left ventricular native T1 values and myocardial ECV. Additionally, being a single-center retrospective investigation with a relatively modest sample size may affect the statistical relevance and generalizability of our results. Looking forward, with the augmentation of sample sizes and further refinement in techniques, the role of CMR functional parameters in heart failure diagnosis is anticipated to become increasingly definitive and pivotal.

In conclusion, this study highlights the diagnostic value of native T1, ECV, and myocardial strain parameters in identifying HFpEF. Particularly, native T1 demonstrates superior diagnostic efficiency compared to ECV, making it a valuable tool. These quantitative indices provide crucial insights into myocardial fibrosis and cardiac function impairment in early-stage HFpEF, facilitating better clinical management and treatment strategies for patients.

## Data Availability

The data analyzed in this study is subject to the following licenses/restrictions: Data from a single center at our institution. Requests to access these datasets should be directed to Yanhui Hao, 1311474058@qq.com.
